# A qualitative inquiry on the status and adequacy of legal instruments establishing infectious disease surveillance in Nigeria

**DOI:** 10.11604/pamj.2018.31.22.14119

**Published:** 2018-09-11

**Authors:** Olusesan Ayodeji Makinde, Clifford Obby Odimegwu

**Affiliations:** 1 Demography And Population Studies Program, Schools of Public Health and Social Sciences, University of The Witwatersrand, Johannesburg, South Africa; 2 Managing Partner, Viable Knowledge Masters, 22 Olusegun Obasanjo Street, Peace Court Estate, Lokogoma, Abuja Federal Capital Territory Nigeria

**Keywords:** Disease outbreaks, infectious diseases, legislation, Nigeria, regulation, surveillance

## Abstract

**Introduction:**

The threat of devastating disease outbreaks is on the rise with several outbreaks recorded across the world in the last five years. The intractable Ebola Virus Disease outbreak in West Africa which spread to Nigeria was a reawakening point. This study aims to review the status and adequacy of the legal framework for disease surveillance in Nigeria. Methods: a mixed methods approach comprising of document reviews and key informant interviews was used in data collection.

**Methods:**

A mixed methods approach comprising of document reviews and key informant interviews was used in data collection.

**Results:**

Fourteen key informants from the federal ministry of health (FMOH) and six States were interviewed. Five legal instruments were identified and reviewed. The Quarantine Act of 1926 remains the active National Law on disease surveillance in Nigeria. An Integrated Disease Surveillance and Response Policy (IDSR) was developed in 2005 as the means for achieving the International Health Regulations (IHR). All six states claimed to have adopted the national IDSR policy though none could present a domesticated version of the policy. Key informants were concerned that Nigeria does not yet have an adequate legal framework for disease surveillance.

**Conclusion:**

The legal instruments establishing disease surveillance in Nigeria require strengthening and possibly enactment as a National Law in order to address emerging disease threats.

## Introduction

Infectious disease outbreaks have been known to occur for ages with devastating impact. In 541 AD, grain merchants' from Egypt transported rats infested with an unknown organism at that time into the Eastern Roman Empire causing the Plague of the Justinian period [1]. This outbreak left over 30 million people dead with significant economic impact [1]. Likewise, the black plague of the 14^th^ century was noted to be responsible for the death of 30 percent of Europe's population, estimated at between 75 and 200 million people [2]. Early in the 19^th^ century and long before the development of the microscope, an outbreak of another unknown disease in London with a high fatality was traced by John Snow to the water pumps, after he was able to map clustering of cases around some particular pumps [3]. He contained the epidemic by removing the handles of the water pumps stopping people from getting water from the contaminated sources. Lately, emerging and re-emerging infectious diseases are posing threat to human populations with fear of outbreaks of these diseases spreading globally and causing significant morbidity and mortality. The Human Immunodeficiency Virus which was first identified in the 1980s is now a pandemic which though has been well curtailed in its acute form by drugs, remains without a cure [4]. The World Health Assembly (WHA) in 1969 adopted the International Health Regulations (IHR) as its legal instrument for implementing its constitutional responsibility for controlling the international spread of infectious diseases. The IHR was an evolution of the International Sanitary Regulations previously adopted by the fourth WHA in 1951. The IHR was revised in 2005 with the purpose and scope “to prevent, protect against, control and provide a public health response to the international spread of disease in ways that are commensurate with and restricted to public health risks and which avoid unnecessary interference with international traffic and trade” [5]. Several African countries including Nigeria implement the Integrated Disease Surveillance and Response (IDSR) strategy as their means for meeting the IHR [6]. The IDSR is a strategy that was first proposed in 1998 by the World Health Organization (WHO) Regional Office for Africa as a framework for a coordinated and integrated surveillance and response.

Nigeria has a population of about 180 million people with limited resources to tackle all her health challenges. As a result, health indices in the country are not among the best in the world. Life expectancy at birth is 54.5 years which is lower than the regional average (60 years) and far lower than the global average (71.4 years) [7]. Various factors including mortalities arising from infectious diseases of public health significance contribute to the low life expectancy in Nigeria. Pneumonia and other neonatal infections remain major killers among children while perinatal infections also contribute significantly to maternal mortality. The country has been affected by the outbreak of various communicable diseases in the past. Some notable ones include the Lassa fever outbreak in the 70's, Yellow fever in the 80's, the Avian influenza outbreak of the early 2000s, recurrent Cerebrospinal Meningitis and recently the Ebola Virus Disease (EVD) outbreak following an imported case in 2014 [8-12]. Polio which was also thought to have been at the verge of elimination has again reemerged [13]. The ability to identify and respond to outbreaks promptly thereby limiting their impact rests on the availability and implementation of appropriate laws, policies, processes and systems to help detect the outbreaks early and take action that will mitigate the spread of such infectious diseases. However, the reliability of surveillance systems in the country in this regard has been questioned [10, 14, 15]. Following the recent EVD outbreak, concerns about the adequacy and out-datedness of laws for disease surveillance in the country have been raised [16]. Thus, it is necessary for an inquiry to be carried out to investigate the adequacy of the legal instruments that guide the surveillance system in the country. The study examines the status and adequacy of national and sub-national legal instruments that govern disease surveillance in Nigeria. The knowledge generated will provide policy makers, public health experts and researchers information useful in advocating for improvement in the legal framework for disease surveillance in the country.

## Methods

This article reports part of a larger research effort to investigate the compliance with disease surveillance and notification by private healthcare providers in Nigeria and the factors that may be affecting their performance. A brief description of the entire study has been previously published [17]. The objectives of the entire study are: to examine the legislative/legal framework for routine disease reporting in Nigeria (nationally and sub-nationally) and how it might affect compliance by private providers; to determine the level of reporting of notifiable diseases by private providers, the completeness of information and how these compare with the public sector; to determine the knowledge and perceptions of private healthcare providers on the importance of routine disease reporting in Nigeria; to identify the barriers to routine disease reporting by private healthcare practitioners/facilities in Nigeria. This article addresses the first objective of the study and employed document reviews and key informant interviews (KII) as the data collection method. A KII guide was developed to drive the interactive sessions. All key informants were interviewed by the principal investigator and the interviews took place between August 2016 and July 2017. Nigeria is a Federation of 36 States and the Federal Capital Territory (FCT). These states and the FCT are grouped into six geopolitical zones of between five and seven States in each zone (North West, North East, North Central, South West, South East and South-South). Of the six geopolitical zones, the South West has the largest concentration of private healthcare facilities of 38% (4338 /11395) based on the 2011 Master Health Facility List [18]. This geopolitical zone was purposively selected for an in-depth investigation based on the main objective of the larger study to focus on private health facilities. Within the South West, a total sample of all the states Lagos, Oyo, Osun, Ogun, Ekiti and Ondo States was included in the study.

Key government officers with responsibilities for disease surveillance and the National Health Management Information System (NHMIS) at the national level and in the six selected States were targeted for interview. The national IHR focal person and the NHMIS officer were identified as key informants at the national level. In each State, the State epidemiologist and the State Health Management Information System (HMIS) officer were identified as key informants. This was a total sample since each State has only one State Epidemiologist and one HMIS officer. The State epidemiologists are responsible for monitoring of all infectious diseases and events of public health importance in the State and manage the State IDSR program while the State HMIS officers are responsible for managing the NHMIS in their State. The KII guide was made up of eight exploratory questions. The guide covered questions on the processes in place for meeting the IHR in Nigeria and in each State; laws and policies in place to ensure compliance with the IHR, contribution of private health facilities to healthcare delivery and the incorporation of private healthcare facilities in the laws on disease surveillance in the country. It also included questions on the perceptions of respondents on the performance of the IDSR and data use. Legal instruments establishing disease surveillance (Laws and Policies) were initially identified by the researchers during the document review and probed for during the KII. Additional documents were identified during the KII. Those identified during the KIIs were also retrieved and reviewed to understand their content and shortcomings. Ethical Approval for the study was obtained from the National Health Research Ethics Committee in the Federal Ministry of Health, Abuja, Nigeria (NHREC Approval Number NHREC/01/01/2007-18/03/2016) and the Wits Human Research Ethics Committee (Non-Medical) of the University of the Witwatersrand, Johannesburg, South Africa (Approval number H16/05/09). In addition, approvals were obtained from the Honorable Minister for Health before national officers were interviewed and from the Commissioners of Health in each State before going ahead with the study in their State. Furthermore, written consent was also obtained from the officers before they were interviewed and this followed the provision of detailed information on the purpose of the study. A participant information sheet which explains the goal of the study and expected outcome was shared with each participant ahead of the interview. The KIIs were recorded using a digital voice recorder and subsequently transcribed verbatim. The transcripts were then used to generate themes based on the objectives of the study. Coding and analysis was manually done by the principal investigator.

## Results

Different legal instruments establishing or reinforcing disease surveillance in the country were identified during the study. Some of the legal instruments were identified during the literature reviews and before the KIIs while some were identified and/or reinforced during the KIIs. In total, six documents were identified and subsequently retrieved and studied. Fourteen key informants were interviewed during the period: two officers from each State (State epidemiologist and State HMIS officer) across the six States of investigation, the national IHR focal person and the NHMIS officer. The interviews lasted between 20 and 45 minutes. The identified documents during this process are presented in Table 1.

**Table 1:** Legal instruments on disease surveillance in Nigeria

	Legal Instrument	Year	Purpose
1	Quarantine Act	1926	The purpose of the Quarantine Act was to “provide for and regulate the imposition of quarantine and to make other provisions for preventing the introduction into and spread in Nigeria, and the transmission from Nigeria, of dangerous infectious diseases
2	National Policy on IDSR in Nigeria	2005	IDSR was adopted as the means of achieving the IHR in 1998 following a decision reached at a WHO Regional Committee for Africa meeting in Zimbabwe but did not come into effect in Nigeria until 2005. The IDSR policy was developed to guide and provide the necessary environment for the planning, implementation, monitoring and evaluation of an IDSR by all tiers of the government including parastatals, private health sector, non-governmental organizations and partners.
3	Technical Guidelines for IDSR in Nigeria	2013	Technical guideline for the implementation of the IDSR was developed in 2013 and followed the international guidelines released by WHO Regional Office for Africa three years earlier.
4	National Health Act	2014	The National Health Act of 2014 is a health law enacted to strengthen the national health system.
5	National Health Information System Policy	2014	The National Health Information System Policy (2014) came into being as a revision of the National Health Management Information System Policy of 2007. It was developed to provide guidance for strengthening of the HIS in the country.
6	Bill for an Act to Establish the Nigeria Public Health Act	2004	The Bill seeks to establish a Public Health Emergency Planning Commission and to repeal the Quarantine Act of 1926

**Legal instruments on disease surveillance in Nigeria:** This section provides a brief summary of the identified legal instruments for disease surveillance in the country.

**The quarantine act:** The Quarantine Act of 1926 remains the active law governing public health in emergencies in Nigeria today [19]. The named diseases in the document include Small Pox. The penalty allotted to defaulters of the Act is N200 (0.7 US Dollars) fine or six months jail time.

**The national policy on IDSR and technical guidelines for IDSR:** The IDSR policy of 2005 is the most recent comprehensive legal instrument reinforcing disease surveillance in Nigeria. The IDSR was adopted to replace the Disease Surveillance and Notification system which had been put in place following a Yellow fever outbreak in 1986/87. The adoption of the IDSR was believed to be the needed change to revamp the ability to predict and detect disease outbreaks in the country. The policy noted that it would be reviewed every five years or as deemed fit by the Minister of Health in consultation with the National Council on Health. It identified the need for the coordination with the NHMIS to avoid duplication. The technical guidelines listed the 41 diseases and conditions of importance to be tracked in Nigeria. It also contained samples of the forms to be used in tracking infectious diseases in the country.

**The national health act:** The National Health Act is not focused on disease surveillance but provides some further support towards the implementation of the NHMIS. Part IV section 35 subsection (1) states that “The Federal Ministry of Health shall facilitate and coordinate the establishment, implementation and maintenance by State ministries, local government health authorities and the private health sector of the health information system at the national, State and local government levels in order to create a comprehensive NHMIS.” It further goes on to provide the Minister with the power to determine the class of data that can be collected and the need for the Minister and State Commissioners to publish annual reports on the health of the citizenry.

**National health information system policy:** The policy prescribes a health data governance structure for the country with the Minister as the chair of the National Health Data Governance Council (NHDGC). The heads of all the health data generating institutions in the country including the National Population Commission, the National Bureau of Statistics and the different departments in the Ministry are members of the NHDGC. It also proposes the creation of State Health Data Governance Councils to be chaired by the Honorable Commissioner of Health in each state with similar representativeness of members across the data generating units in the State. The policy was designed to drive a good coordination and governance of the health information system.

**The national public health bill:** Though it was discovered that a Public Health Bill had been waitlisted since 2004 in the Nigerian Senate for possible enactment as a Law, this Bill is yet to be ratified by legislators [20].

**Key informant's perspectives on legal instruments:** The key informants interviewed buttressed the finding from the document reviews that the IDSR policy of 2005 is the most recent comprehensive legal instrument guiding disease surveillance in the country. A key informant noted that “States actually agreed at the National Council on Health (NCH) years back to implement the IDSR strategy and that is what we have on ground”. The NCH is the highest policy making body in Nigeria with regards to health and is made of the Minister for Health, Minister of State for Health, the Commissioners of Health across the 36 states of the Federation, the Secretary of Health and Human Services of the Federal Capital Territory and the Permanent Secretary at the Federal Ministry of Health [21]. Besides the IDSR policy, another legal instrument identified by key informants was the National Health Act. An informant noted that “We are capitalizing on the National Health Act. The Act has a section on disease surveillance”. Several key informants held opinions that other laws governing infectious diseases were as old as the country and not useful with current disease challenges. It was stated by a key informant that “The public health laws are already outdated. They were drafted back in 1958.” Another informant noted that “The public health laws in Nigeria are old and the penalties spelt out in them are so meager (five Naira), as such, it is not relevant to deter offenders.” A key informant highlighted that small pox which was eradicated in 1980 remains one of the diseases highlighted in the main public health law in the country which makes it archaic. Key informants from the States noted that their States were aligning with the Federal Governments' policy on infectious disease control. One informant highlighted that “There is no specific law or guideline apart from the Federal Ministry of Health document on the IDSR. This is what has been adopted in our state.” An informant opined that “We have adequate laws that can help us in meeting the IHR. However enforcing their implementation is the problem.” Contrary to this view, most informants do not believe that Nigeria and their specific states (for the state officers) have adequate laws on disease surveillance. They believe that a revision of existing laws was necessary to address emerging issues. Notwithstanding, they all agreed to the poor implementation of existing laws. It was noted by an informant that there were ongoing efforts to strengthen legislation on infectious disease control in his State. He noted that following the EVD outbreak of 2014, a Bill to enact a law on cremation of suspected infectious disease cases has been under discussion in the legislature. However, progress to enact the law has been slow. This draft Bill was not available to the research team upon request as it was stated to be a confidential document.

## Discussion

Nigeria is a Federation with three tiers of government: the federal government, state governments and the local governments [22]. The Federal Government is responsible for the national administration, State Governments oversee the states and Local Government Authorities oversee the smaller local government areas within the states. Constitutional responsibilities on health cut across the three tiers of government [22,23]. Laws, policies and guidelines at the Federal level do not necessarily bind the States to implement them unless they are first adopted as a State law. Since the return of Nigeria to democratic governance in 1999, several health-related laws have been enacted by the federal government some of which have been ratified by the State legislatures following modifications. Such includes the Child Rights Act 2003 which has been ratified in some States with variations to the original law passed at the national level [23]. The National Health Act of 2014 also makes extensive provision for States to enact laws within their territories to govern their health system [21]. Thus from practice, it can be inferred that national laws on health do not necessarily bind the States. Two classes of legal instruments were identified in the course of the study and these are: Laws and Policies. Laws are the higher of the two having been ratified by legislators in parliament before being signed into Law by the president or a State governor. On the other hand, policies are of lesser authority and are developed for specific purposes often by civil servants in the ministries and do not necessarily undergo the rigor of review, adoption and ratification by legislators. Non-compliance with Laws can be regarded as a violation with potential consequences that can include criminal charges and jail time while non-compliance with policies does not necessarily confer the same status. More so, policies can be changed easily by successive governments threatening their continuity and ability to achieve their goals [24]. Multiple cadres of legal instruments are also available at the WHA (Convention agreements, Regulations and Recommendations) and have different uses and authorities [25]. Previous studies have revealed inadequate capacity among policymakers to transform research to policies and laws in Nigeria worsened by poor knowledge of researchers to adequately understand health policy needs and tailor their studies to address the knowledge gap [26, 27]. Such poor capacity among policy and lawmakers might be responsible for the little attention given to the enactment of laws targeted at Global Health Security within the country's territory.

The Quarantine Act and the National Health Act are the current Laws that govern disease surveillance in the country. However, it is archaic having been established as a Law in 1926, several decades before the IHR came into effect. One of the diseases identified in the document was eradicated over 35 years ago, highlighting the need for a revision. The National Health Act which was signed into Law in 2014 identified the need for the strengthening of the NHMIS in order to be able to deliver on its mandate. The level of detail in the National Health Act for disease surveillance is considered inadequate by most respondents as it was not enacted for this purpose. The availability of various legal instruments at the national level for different diseases and conditions do not necessarily mean that these are being adhered to in the States which are a different administrative level of government. The states are independent and thus, ratification is necessary where the Law is not on the exclusive list of issues addressed solely by the Federal Government. This is similar to the concern raised by Gostin and colleagues (2017) where they made an assertion that development of global legal instruments does not mean they are being implemented by member states. Responsibility still rests on member states to adopt them and their inaction cannot be easily sanctioned since there is no law enforcement body to prosecute defaulters. Strategies that are being used to make the states more responsive to federal laws include incentivizing for results as is seen on the World Bank funded Saving One Million Lives-Program for Results [28]. However, the effectiveness of this strategy still needs further evaluation. Many of the respondents do not think that Nigeria has adequate laws on disease surveillance which is worsened by the poor implementation of existing laws. This has also been echoed in the literature following the EVD outbreak of 2014 [16]. A Public Health Bill was discovered to be awaiting assent in the Nigerian Senate since 2004. This Bill sought to establish a Public Health Emergency Planning Commission with the hope that this institution will improve the responsiveness of the system to public health emergencies. The long delay in finalizing this Bill is a huge challenge to the health sector. However, it was also observed that several other Bills were awaiting review. While the establishment of an agency may provide some opportunities for structured response to infectious diseases, the unavailability of adequate Laws for the system will make it unable to achieve its goal. A Nigeria Center for Disease Control (NCDC) supported by the US Centers for Disease Control and Prevention (CDC) has been in operation since 2012 [29]. However, this operated as a US CDC supported project of the government. Early in 2017, the Federal Executive Council (chaired by the President) approved a memo to adopt the NCDC as a standalone government institution and a request has been sent to the legislature for ratification [30]. It is unclear if the effort to establish the NCDC will nullify the public health bill which has been awaiting reading on the floor of the Senate since 2004. Notwithstanding it is noteworthy that information on the NCDC first surfaced in 2008 [29]. The long delay before it received attention is a concern that must be addressed. The IDSR strategy which was developed to implement the IHR is still governed by a policy, the lesser of the two legal instruments. The Quarantine Act predates the IDSR policy and both documents do not necessarily align on diseases that are to be tracked. While many officers interviewed generally agreed that they were implementing the national policy on IDSR since it was adopted at a NCH meeting several years earlier, none could show any document ratifying the IDSR by their State legislature or detailing how IDSR should be implemented in their State by their Ministry of Health. This is a major shortcoming as it has been established that national laws and policies on health do not necessarily bind the States. As such, State specific Laws or policies addressing disease surveillance are necessary to strengthen the commitment towards disease surveillance in each State.

## Conclusion

The legal framework establishing disease surveillance in Nigeria requires strengthening and possible enactment as a law to give it the authority it deserves as well as to update it to address emerging Global Health Security challenges. State governments should domesticate policies or laws on disease surveillance to demonstrate buy-in and improve their commitment to its implementation. Lawmakers and Policymakers need to be educated on the importance of prompt attention to laws on Global Health Security.

### What is known about this topic

- The threat of devastating disease outbreaks is on the rise globally and countries need an efficient surveillance system for detecting and responding to outbreaks to mitigate their impact;
- The first step towards a reliable system is a legal or regulatory framework that establishes and enforces the surveillance system in a country;
- Disease surveillance is poor is several low and middle income countries including Nigeria and efforts are required to improve their performance.

### What this study adds

- This study provides a snapshot of the status and the adequacy of the legal and regulatory framework which establishes disease surveillance in Nigeria;
- It also highlights concerns by key stakeholders that Nigeria does not have an adequate legal or regulatory framework for addressing disease surveillance and those currently available are not being implemented.

## Competing interests

The author declare no competing interest.
